# Supplementary material for: Sink Strength Promoting Remobilization of Non-Structural Carbohydrates by Activating Sugar Signaling in Rice Stem during Grain Filling
Source: Int J Mol Sci. 2022 Apr 27;23(9):4864. doi: 10.3390/ijms23094864 (PMC9106009; doi:10.3390/ijms23094864)
Supplement: Supplementary file 1 [file ijms-23-04864-s001.zip › ijms-1689208-supplementary.pdf]

Supplementary data

Table S1. Sequences of primers for Actin and genes for qRT-PCR

Gene	Forward primer 5′ → 3’	Reverse primer 5′ → 3’
Actin	CAATCGTGAGAAGATGACCC	GTCCATCAGGAAGCTCGTAGC
OsSUS2	GAGGCTGATGACCTTGACTGG	CCCTCCATTACTTGGATGTGCT
OsAGPL1	GGAAAGGTTCCTATTGGAATCG	GGAGGGCTTTATTCCACCTCAG
α-Amy3	GATCCCGAACGGTGGAAGG	GAACCGGGAGGTCTCATGTC
OsTPS8	TTAATCCTCAGGGTGTGGGC	CAGGCGAAGGGCTGATCATA
OsTPP1	GTTGGGCGGCCTGCCTTCAA	AGCCTCCTGCGAGGTGACGA
OSK1	AACCAGAGGTAACAGGCAGG	CATCTGTCAAGGAATGCAGG
OSK24	GCAGTGATCCTCATGCCAG	CCTTCGCTGTCTAAGGGACT

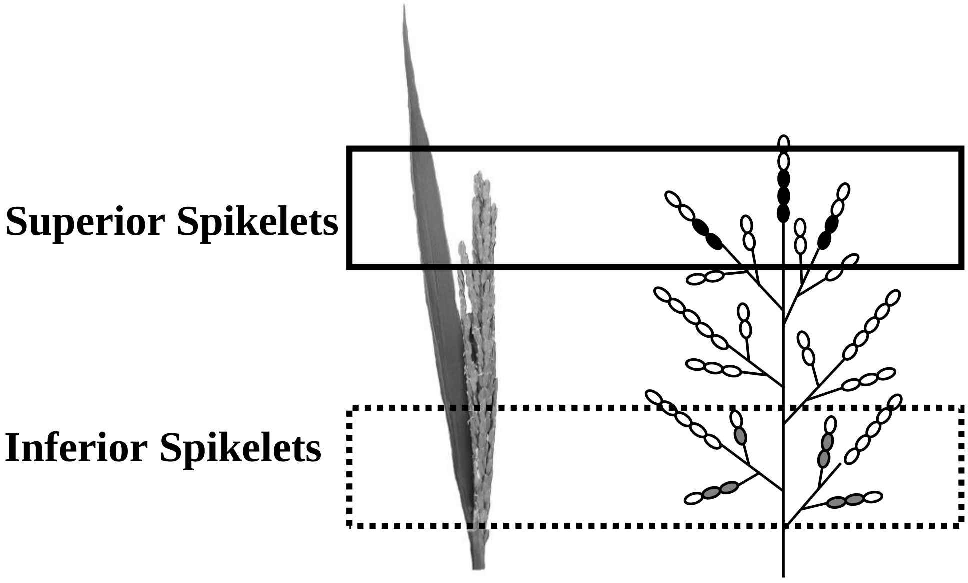

Supplementary Figure S1. Schematic diagram of rice panicle structure aookied to study the effect of flowering time gap and competition between superior spikelets and inferior spikelets in rice.
